# Multi-output learning for systematic missing value imputation in DNA methylation arrays

**DOI:** 10.1093/bioadv/vbag052

**Published:** 2026-02-15

**Authors:** Tao Ma, Jinfu Nie, Jian Huang, Yong-Biao Zhang, Joanna M Biernacka, Liguo Wang

**Affiliations:** Division of Computational Biology, Department of Quantitative Health Sciences, Mayo Clinic College of Medicine & Science, Rochester, MN 55905, United States; Hefei Cancer Hospital of CAS; Institute of Health and Medical Technology, Hefei Institutes of Physical Science, Chinese Academy of Sciences, Anhui 230031, China; School of Engineering Medicine, Beihang University, Beijing 100191, China; School of Engineering Medicine, Beihang University, Beijing 100191, China; Division of Computational Biology, Department of Quantitative Health Sciences, Mayo Clinic College of Medicine & Science, Rochester, MN 55905, United States; Division of Computational Biology, Department of Quantitative Health Sciences, Mayo Clinic College of Medicine & Science, Rochester, MN 55905, United States

## Abstract

**Motivation:**

Illumina DNA methylation arrays have evolved rapidly, expanding genomic coverage while introducing backward incompatibilities by removing many CpG sites present in earlier versions. These changes result in systematic missing values when integrating data across array generations and substantially limiting the reuse of legacy datasets.

**Results:**

We developed a two-stage framework for imputing missing DNA methylation values. The procedure first imputes randomly missing values using standard imputation techniques and then addresses systematic missingness using multi-output machine learning models, including support vector regression, nearest-neighbor methods, random forest models, and deep neural networks. When evaluated on real datasets with up to fifty percent induced missingness, the proposed framework consistently outperformed conventional imputation approaches. It also accurately imputes the missing CpG sites between methylation arrays and reduced representation bisulfite sequencing data, enabling robust cross-platform data integration. Analyses of large brain tumor methylation datasets demonstrate that the method restores array-specific methylation patterns while preserving biological complexity. Importantly, imputing missing methylation sites significantly improves the performance of epigenetic age prediction models.

**Availability and implementation:**

This tool is implemented in the Python package “ultra-impute,” freely available at https://github.com/liguowang/ultra-impute. A code snippet demonstrating the usage of the ultra-impute package is provided in a Jupyter Notebook (https://github.com/liguowang/ultra-impute/blob/master/doc/Tutorial.ipynb).

## 1 Introduction

While sequencing-based DNA methylation profiling techniques, such as reduced representation bisulfite sequencing (RRBS) and whole-genome bisulfite sequencing (WGBS), have gained popularity for studying methylomes, array-based DNA methylation assays remain the preferred choice in many scenarios. First, methylation arrays only target a subset of biologically significant CpGs, making them cost-effective and scalable for large studies, such as epigenome-wide association studies (EWAS). Second, data generated from methylation arrays require less computational and bioinformatic efforts, simplifying downstream analyses. Lastly, their standardized design facilitates data integration across different studies, enhancing their utility in training machine learning and AI models (e.g. epigenetic clocks) that need large and diverse datasets.

**Figure 1:**
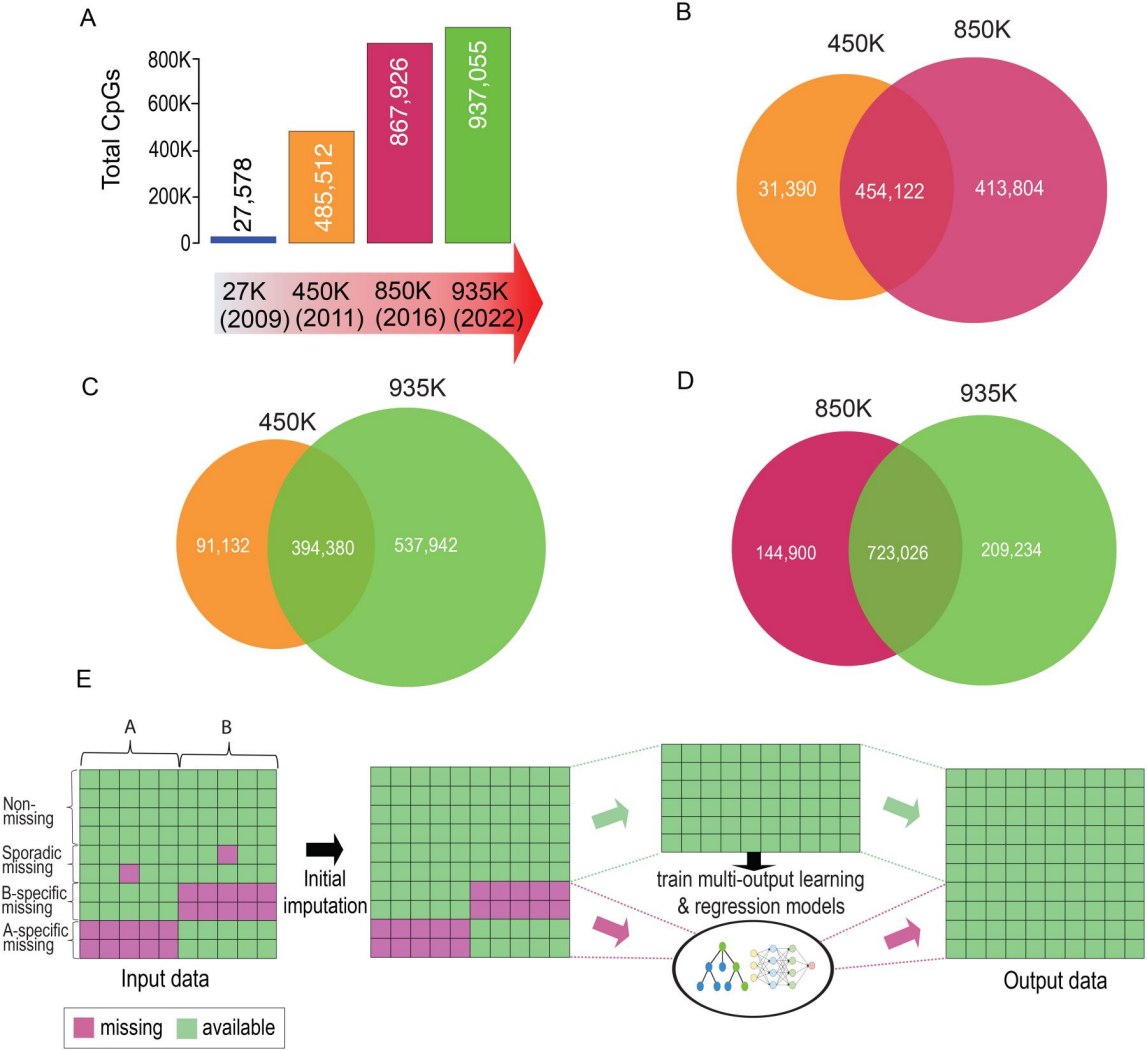
Overview of the systematic missing values in Illumina DNA methylation arrays and the MOREL imputation method. (A) The coverage and evolution of Illumina genome-wide DNA methylation arrays. 450K, Infinium HumanMethylation450 BeadChip; 850K, Infinium MethylationEPIC v1.0; 935K, Infinium MethylationEPIC v2.0. (B) – (D): Venn diagrams show the incompatibilities (systematic missing) between methylation arrays. (E) Flowchart shows the procedure of MOREL: (1) Samples are assigned into two groups (termed as A and B) using K-means clustering or user provided group designations. (2) CpGs are divided into four groups including “Non-missing,” “Sporadic missing,” “A-specific missing” and “B-specific missing.” Sporadic missing values (if any) are filled using canonical imputation methods. (3) multi-output learning or regression models training. (4) structural missing values are filled using MOREL methods.

Illumina’s methylation arrays have evolved from Infinium HumanMethylation27 (27K) to Infinium HumanMethylation450 BeadChip (450K), MethylationEPIC BeadChip v1 (850K), and most recently, MethylationEPIC BeadChip v2.0 (935K) (Fig. 1A). While newer versions of the arrays target more CpGs, they also remove a significant number of CpGs prone to producing inconsistent results due to cross-hybridization or mapping to polymorphic regions. This leads to systematic missing values, which complicate longitudinal data integration across different array generations (Fig. 1B–D). For example, The Cancer Genome Atlas (TCGA) consortium profiled DNA methylation in 2728 tumor samples using the 27K array and 9639 samples using the 450K array. Since restricting analyses to shared CpGs results in substantial data loss, integrating data generated from current arrays with these valuable legacy datasets remains a major challenge.

Traditional imputation approaches, such as iterative imputation (e.g. Multiple Imputation by Chained Equations, or MICE (van Buuren and Groothuis-Oudshoorn 2011)), often struggle with datasets containing a large number of missing values for several reasons. First, most traditional approaches assume missing at random (MAR), making them ineffective when dealing with structured missingness (i.e. many CpGs are completely missing from a subset of samples); e.g. imputation algorithms may fail to converge between suboptimal imputations. Second, traditional methods rely heavily on the presence of complete or partially complete data to estimate missing values. When a large portion of the data is missing, the initial estimates are often inaccurate, affecting subsequent imputations because of error propagation. Third, linear regression-based methods may fail to accurately capture complex or nonlinear relationships, especially when the observed data are sparse due to extensive missingness.

To address these challenges, we proposed the Multi-Output Regression and Learning (MOREL) approach. MOREL treats the imputation of multiple missing values as a joint prediction problem, leveraging relationships among variables to improve accuracy. By simultaneously predicting multiple missing variables while accounting for their interdependencies. MOREL provides many advantages. First, it captures the relationships among the target variables, ensuring that imputations are consistent and reflect their underlying structure. Second, it improves accuracy by utilizing shared information across variables, which is particularly beneficial when outputs are highly correlated—such as CpGs located within the same genomic features (genes, promoters, CpG islands) that tend to co-methylate. Lastly, MOREL ensures that the imputed values are mutually plausible, particularly in datasets with complex variable interactions, making it an efficient and reliable approach for imputing missing data.

## 2 Methods

### 2.1 Implementation of the ultra-impute package

The ultra-impute package (https://github.com/liguowang/ultra-impute) is implemented using the Python programming language. Numerical computations and data manipulation are built upon SciPy (https://scipy.org/), NumPy (https://numpy.org/), and pandas (https://pandas.pydata.org/). Deep neural network implementations are built on the TensorFlow (https://www.tensorflow.org/) and Keras (https://keras.io/) frameworks.

The ultra-impute package includes four MOREL approaches: random forest (MOREL-RF), support vector regression (MOREL-SVR), k-nearest neighbor (MOREL-KNN), and deep neural network (MOREL-DNN). MOREL-RF is based on the “RandomForestRegressor” function from “sklearn.ensemble,” wrapped with “MultiOutputRegressor.” Similarly, the MOREL-SVR is built upon the “LinearSVR” function from the “sklearn.svm,” and the MOREL-KNN is built upon the “KNeighborsRegressor” function from “sklearn.neighbors.” For the MOREL-DNN algorithm, a sequential model was built with an input layer, a hidden layer, and an output layer. The number of neurons in the input and output layers equals the number of samples with non-missing and missing values, respectively. In the hidden layer, the number of neurons is empirically set to the mean of the neurons in the input and output layers, the “kernel_initializer” is set to “he_uniform,” and the activation is “RELU.” In the output layer, the “kernel_initializer” is set to “he_uniform” and the activation is “linear.”

Our MOREL imputation process involves the following four key steps (Fig. 1E).

1. Identification of sample groups. We used the *K*-means clustering to group samples after binarizing all DNA methylation beta values (assigning “1” to non-missing values and “0” to missing values). The purpose of binarization is to enable samples to be grouped by the probes’ presence-absence status rather than the reported DNA methylation beta values. At the current version, only *K* = 2 is supported, meaning that all samples will be clustered into two groups (mimicking the integration of two different types of arrays). *K*-means clustering could accurately identify samples with data generated from different arrays, but this step can be overridden by user-provided group designations.
2. Categorize CpGs. After samples are assigned to two groups (termed as group “A” and “B” hereafter for simplicity), all probes (i.e. CpGs) will be divided into four categories: “Non-missing,” “Sporadic missing,” “A-specific missing,” and “B-specific missing.” “Non-missing” refers to CpGs without any missing values in either “A” or “B.” “Sporadic missing” refers to CpGs with random missing values in either “A” or “B,” but the pattern of missing is not group specific. “A-specific missing” and “B-specific missing” refer to structural (systematic) missingness from array incompatibility. If there are CpGs with all values missing from both groups, they will be removed or kept as is.
3. Imputation of sporadic missing values. “Sporadic missing” values are imputed using standard methods, including IterativeImputation, Random Forest, K-Nearest Neighbors, and Buck’s method. Based on our evaluation, these methods are generally effective when the proportion of missing values is low.
4. Imputation of systematic missing values. Systematic missing values are imputed using MOREL techniques, including MOREL-RF, MOREL-SVR, MOREL-KNN, and MOREL-DNN. Although RF and KNN are applied to both sporadic and systematic missingness, their implementations differ. For instance, sporadic missing values are imputed using a single trained RF model, whereas systematic missing values require fitting a separate RF model for each target. When *K* = 2, two distinct DNN models are trained to predict “A” missing values from “B” and *vice versa*.

In addition to the four MOREL approaches, ultra-impute integrates imputation algorithms from several widely used Python libraries, including impyute (https://github.com/eltonlaw/impyute), MissForest (https://github.com/yuenshingyan/MissForest), fancyimpute (https://github.com/iskandr/fancyimpute), and scikit-learn (https://scikit-learn.org/). A total of 11 other commonly used methods, along with the MOREL approaches, are summarized in Table 1.

**Table 1:** A list of 15 imputation methods implemented in the ultra-impute package.

Function	Description
fill_trend()	Replace missing values using methods such as mean, median, minimum (min), maximum (max), backfill (bfill), or forward fill (ffill).
fill_rand()	Fill missing values with values randomly selected from the same row or column.
fill_mw()	Replace missing values with mean values computed using a moving/sliding window along rows or columns.
fill_fKNN()	Replace missing values with values computed from the weighted mean of K-nearest neighbors (adapted from the impyute library).
fill_KNN()	Replace missing values with values computed as the mean of K-nearest neighbors (same as scikit-learn).
fill_ref()	Replace missing values with values computed as the mean of K-nearest neighbors. Unlike fKNN and KNN, the nearest neighbors are identified from an external reference dataset.
fill_EM()	Impute missing values using the Expectation-Maximization (EM) algorithm.
fill_Buck()	Impute missing values using Buck’s method, a statistical technique for handling incomplete data.
fill_NNM()	Impute missing values using NuclearNormMinimization. Only work for small dataset.
fill_SoftImpute()	Same as scikit-learn’s SoftImpute method.
fill_IterativeSVD()	Same as scikit-learn’s IterativeSVD method.
fill_IterativeImputer()	Same as scikit-learn’s IterativeImputer method.
fill_MatrixFactorization()	Same as fancyimpute’s MatrixFactorization method.
fill_RF()	Imputes missing data using the Random Forest algorithm.
fill_morel()	Imputes missing values using multi-output regression and learning approaches (MOREL-KNN, MOREL-RF, MOREL-SVR and MOREL-DNN).

### 2.2 Evaluation datasets

To evaluate the imputation accuracy of different methods, we obtained DNA methylation datasets from two sources:

1. Whole blood cohort: DNA methylation data from 30 whole blood samples were downloaded from the GEO database (accession numbers from GSM2814050 to GSM2814079).
2. TCGA GBM tumor cohort: DNA methylation data from 30 TCGA GBM tumor samples were downloaded from the GDC Data Portal (TCGA barcodes: TCGA-14-1402-02, TCGA-06-0152-01, TCGA-19-5950-01, TCGA-06-5413-01, TCGA-19-5954-01, TCGA-76-6283-01, TCGA-06-5408-01, TCGA-19-A6J4-01, TCGA-06-5856-01, TCGA-32-1980-01, TCGA-14-0862-01, TCGA-14-1402-01, TCGA-76-6282-01, TCGA-06-A5U1-01, TCGA-RR-A6KB-01, TCGA-06-0152-02, TCGA-76-6286-01, TCGA-76-6664-01, TCGA-19-0957-02, TCGA-74-6577-01, TCGA-76-6193-01, TCGA-06-5859-01, TCGA-87-5896-01, TCGA-06-5411-01, TCGA-14-0740-01, TCGA-14-1450-01, TCGA-76-6657-01, TCGA-06-AABW-11, TCGA-06-6391-01, TCGA-76-6662-01).

DNA methylation data for both cohorts were generated using Illumina’s HumanMethylation450 BeadChip (450K). Within each cohort, samples were randomly divided into two groups of 15. To simulate datasets with varying levels of structural missing, beta values from 10%, 30%, and 50% of randomly selected CpGs were removed from one group only. For example, removing values of 50% of CpGs from one group results in an overall missing level of 25% across the dataset.

To assess the effect of methylation level on imputation accuracy, CpGs with values removed in the 50% scenario were stratified into four tiers (0–0.25, 0.25–0.5, 0.5–0.75, 0.75–1) according to their original mean beta values. Additionally, we performed t-tests on the original beta values between the two sample groups and selected CpGs with adjusted *P*-values < 0.05 to represent a challenging case of highly differentially methylated loci.

To evaluate whether our MOREL method (using MOREL-DNN as an example) could restore structural missing from real data, we downloaded the third cohort, which contains 16 FFPE samples from different types of brain tumors. The DNA methylome of each tumor was profiled using both Infinium MethylationEPIC v1.0 (850K) and Infinium MethylationEPIC v2.0 (935K); therefore, a total of 32 samples were downloaded from GEO (GSE229715) and analyzed.

To access MOREL’s performance beyond the human species and across different platforms, we utilized methylation data from 37 mouse samples profiled using both MMA and RRBS platforms (https://zenodo.org/records/7102827). After merging the datasets, we identified 22 144 shared CpGs and created two modified datasets. In the first dataset, values of 50% of randomly selected CpGs were removed from MMA, allowing them to be imputed later using RRBS data. In the second dataset, we removed the values of the same CpGs from RRBS so they could be imputed from MMA data.

To evaluate whether imputation enhances the performance of DNA methylation–based aging clocks, we analyzed a cohort of 124 healthy human samples with methylation profiles generated from blood using the Illumina MethylationEPIC BeadChip v2.0 (935K) and corresponding chronological age data (unpublished). These healthy individuals serve as a control cohort for bipolar disorder studies at Mayo Clinic. Their chronological ages were used as the benchmark to assess whether epigenetic age predictions from the clocks improved following imputation.

### 2.3 Evaluation metrics

To assess and compare the performance of each imputation method, we computed MAE (Mean Absolute Error), RAE (Relative Absolute Error), RMSE (Root Mean Squared Error), and R^2^ (Coefficient of Determination), which are defined as follows (*y*_i_ represents the actual value, $\bar{y_{i}}$ represents the mean of actual values, $\hat{y_{i}}$ represents the imputed value, SS_res_ represents the “sum of squared residues,” and SS_tot_ represents the “total sum of squares”):


$$\mathrm{MAE}=(\frac{1}{n})*\Sigma \;|y_{i}-\hat{y_{i}}|$$

$$\mathrm{RAE}=\frac{\Sigma |y_{i}-\hat{y_{i}}|}{\Sigma |y_{i}-{\bar{y}}_{i}|}$$

$$\mathrm{RMSE}=\sqrt{(\frac{1}{n})*\sum_{i=1}^{n}{\left(y_{i}-\hat{y_{i}}\right)}^{2}}$$

$$R^{2}=1-\frac{{SS}_{\mathrm{res}}}{{SS}_{\mathrm{tot}}}=\frac{\sum {\left(y_{i}-\hat{y_{i}}\right)}^{2}}{\sum {\left(y_{i}-\bar{y_{i}}\right)}^{2}}$$

## 3 Results

### 3.1 Overview of the ultra-impute package

The ultra-impute package implements the MOREL framework, specifically designed to address systematic or structural missing values when integrating data from different methylation arrays. The MOREL methods comprise four multi-output wrapped approaches: random forest (MOREL-RF), support vector regression (MOREL-SVR), k-nearest neighbor (MOREL-KNN), and deep neural network (MOREL-DNN).

Beyond MOREL, ultra-impute includes a diverse set of standard imputation techniques, such as Iterative Imputation, Expectation-Maximization (EM) (Dempster *et al.* 1977), Nuclear Norm Minimization (NNM) (Gu et al. 2014), SoftImpute (Mazumder *et al.* 2010), Iterative SVD, and Matrix Factorization (Koren *et al.* 2009). These methods predict missing values based on various statistical or machine learning trends, such as computing the mean or median along a specific axis (row or column) or within a sliding window. Trends can also be estimated using the weighted mean of K-nearest neighbors (KNN), where neighbors are selected from either the dataset itself or an external reference. For time-series data, ultra-impute supports both forward filling (using preceding data points) and backfilling (using subsequent data points).

Overall, ultra-impute provides a comprehensive suite of 15 imputation methods, ensuring robust and flexible handling of missing data across a wide range of scenarios (Table 1).

### 3.2 Evaluation of imputation accuracy

We used two public datasets to systematically compare the accuracy of different imputation algorithms: one dataset was generated from whole blood (Sugden *et al.* 2019) and the other one from the TCGA GBM (Glioblastoma Multiforme) brain tumor cohort (Brennan *et al.* 2013). From each dataset, we introduced 50% missingness (i.e. removed methylation beta values from half the samples) across 10%, 30%, and 50% of the CpGs. This simulation mimics a scenario where half of the samples were profiled using one array, while the other half were measured with a different type of array. Various imputation methods were then applied, and their accuracy was assessed by comparing the imputed values with the original values using multiple evaluation metrics, including Mean Absolute Error (MAE), Relative Absolute Error (RAE), Root Mean Squared Error (RMSE), and the Coefficient of Determination (see Section 2).

**Figure 2:**
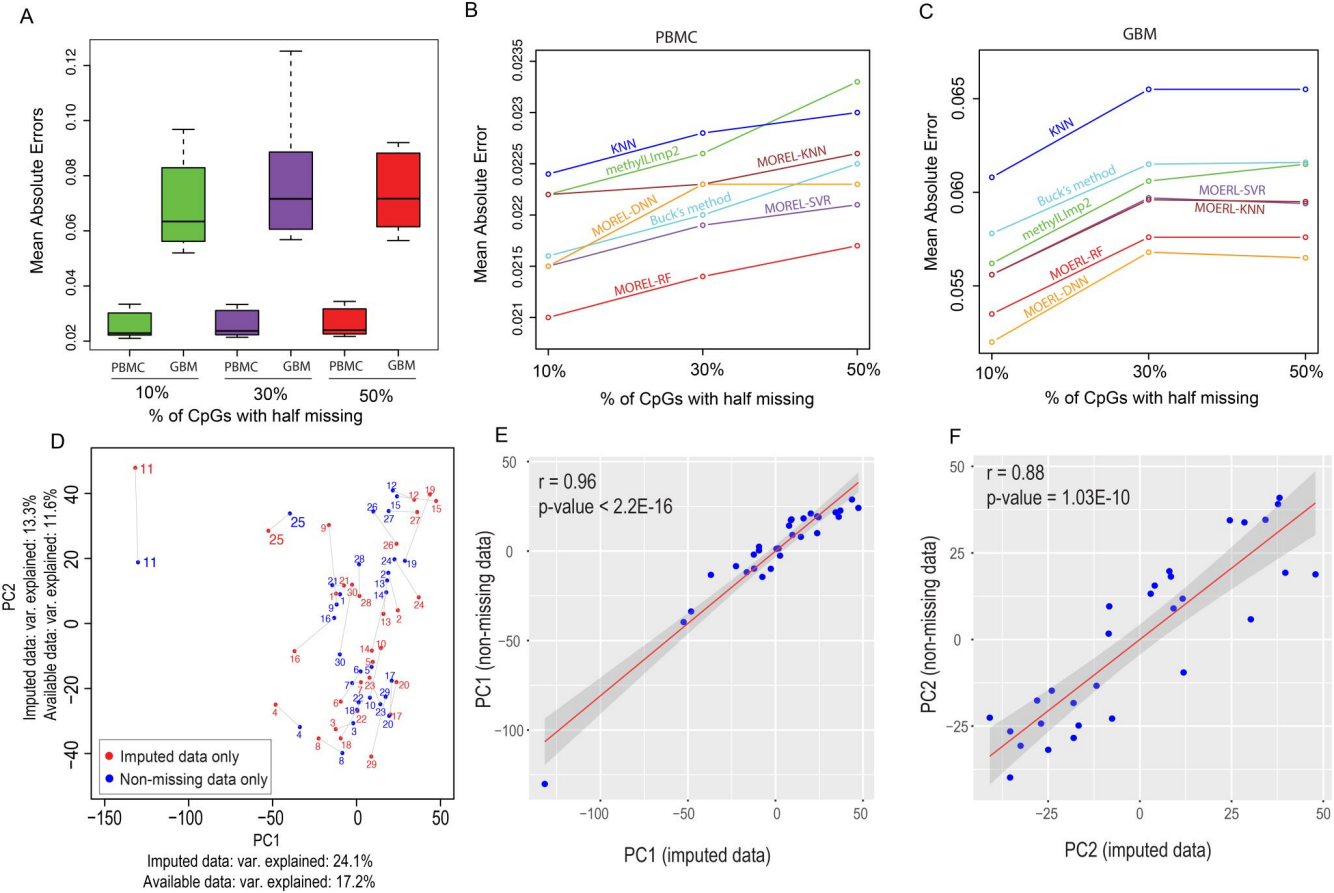
Evaluation of the imputation performance of different algorithms. (A) Comparison of mean absolute errors (MAEs) between whole blood and TCGA GBM datasets. Each dataset contains 30 samples with manually introduced missing values at 5%, 15%, and 25% levels. The y-axis represents the MAEs of 14 imputation methods: “Row Mean,” “KNN,” “EM,” “Buck,” “SoftImpute,” “IterativeSVD,” “IterativeImputer,” “Matrix Factorization,” “RF,” “methylLImp2,” “MOREL-RF,” “MOREL-SVR,” “MOREL-KNN,” and “MOREL-DNN.” (B) MAE comparison of the top seven methods applied to DNA methylation data generated from whole blood. (C) MAE comparison of the top seven methods applied to DNA methylation data generated from TCGA GBM samples. (D) Two-dimensional projections of PCA calculated from original (non-missing) data points (blue) and imputed data points (red). Each of the 30 GBM samples is labeled numerically from 1 to 30. (E) Correlation between the first principal component (PC1) calculated using imputed data only (horizontal axis) and non-missing data only (vertical axis). (F) Correlation between the second principal component (PC2) calculated using imputed data only (horizontal axis) and non-missing data only (vertical axis). Abbreviations: GBM, glioblastoma multiforme; MAE, mean absolute error; KNN, k-nearest neighbors; EM, expectation-maximization; SVD, singular value decomposition; RF, random forest; SVR, support vector regression; DNN, deep neural network; MOREL, Multi-Output Regression and Learning; PC, principal component.

Overall, imputation accuracy was significantly higher in methylation data generated from blood samples (MAE = 0.0191–0.0204), compared to the GBM tumor samples (MAE = 0.0525–0.0567) (Fig. 2A, Table 1, available as supplementary data at Bioinformatics Advances online). This is probably because tumor samples exhibit increased heterogeneity at both the cellular (e.g. tumor purity variability) and genomic levels (e.g. somatic alterations). Among all the tested algorithms, the MOREL framework—comprising MOREL-RF, MOREL-SVR, MOREL-KNN, and MOREL-DNN—outperformed traditional imputation methods such as Iterative Imputer, Iterative SVD, SoftImpute, Buck’s method, Random Forest, KNN, EM, and methyLImp2, a state-of-the-art algorithm specifically designed for imputing missing DNA methylation values (Plaksienko *et al.* 2024) (Fig. 2B and C, Table 1, available as supplementary data at *Bioinformatics Advances* online). Within the traditional methods, Buck’s method, methyLImp2, and KNN performed the best, while Iterative SVD and Iterative Imputer showed the poorest accuracy. Within the MOREL framework, minor performance differences were observed: MOREL-RF achieved the highest accuracy for imputing missing values in whole blood samples, while MOREL-DNN performed best in the more heterogeneous GBM samples (Fig. 2B and C).

To evaluate whether imputed data preserved the original biological complexity, we conducted principal component analyses (PCA) on the GBM dataset with 25% missing values (i.e. 50% of CpGs having missing values in half of the samples). The first PCA analysis was performed using the original data, while the second PCA was applied to the values imputed using the MOREL-DNN method (Fig. 2D). The principal components (PCs) showed strong concordance, with Pearson’s correlation coefficients of 0.96 and 0.88 for PC1 and PC2, respectively (Fig. 2E and F), indicating that MOREL-DNN maintains data complexity and dimensionality. Similar results were observed for other MOREL methods (data not shown).

To examine the influence of methylation levels (measured by beta values) on imputation accuracy, we stratified CpGs into four categories according to their mean beta value across samples (0–0.25, 0.25–0.5, 0.5–0.75, 0.75–1). We found that CpGs with low (0–0.25) and high (0.75–1) methylation levels were imputed more accurately than those with intermediate methylation levels (0.25–0.75) in both blood and GBM samples. This is because CpGs that are consistently unmethylated (*β* = 0–0.25) or consistently methylated (*β* = 0.75–1) often exhibit lower variance across samples. We further evaluated the performance of imputation methods on the differentially methylated CpGs between two groups, which represent challenging cases, and found that MOREL maintained robust and consistently high accuracy even for these difficult loci (Table 1, available as supplementary data at *Bioinformatics Advances* online).

### 3.3 Restoring missing DNA methylation landscapes between 850K and 935K arrays

To further demonstrate the utility of our MOREL approach, we analyzed DNA methylation data from 16 FFPE brain tumor tissues, each profiled simultaneously using the 850K and 935K methylation arrays (Table 2, available as supplementary data at *Bioinformatics Advances* online). We generated a dataset comprising 1 073 899 CpGs, of which 722 359 (67.26%) are shared between the two arrays, while 351 540 (32.74%) are array-specific—207 560 unique to the 850K array and 143 980 exclusive to the 935K array—resulting in systematic missing values for half of the samples in these CpGs. These missing values arise from inherent incompatibility between 850K and 935K and will persist in any data integration involving these two arrays.

We first applied Buck’s method to impute sporadic missing values present in 1713 shared CpGs. Next, we used the MOREL-DNN method to impute methylation values for the systematically missing CpGs. To evaluate its effectiveness, we separated the dataset back into 850K and 935K arrays and performed PCA analyses separately on non-missing CpGs only and imputed CpGs only for both datasets.

**Figure 3:**
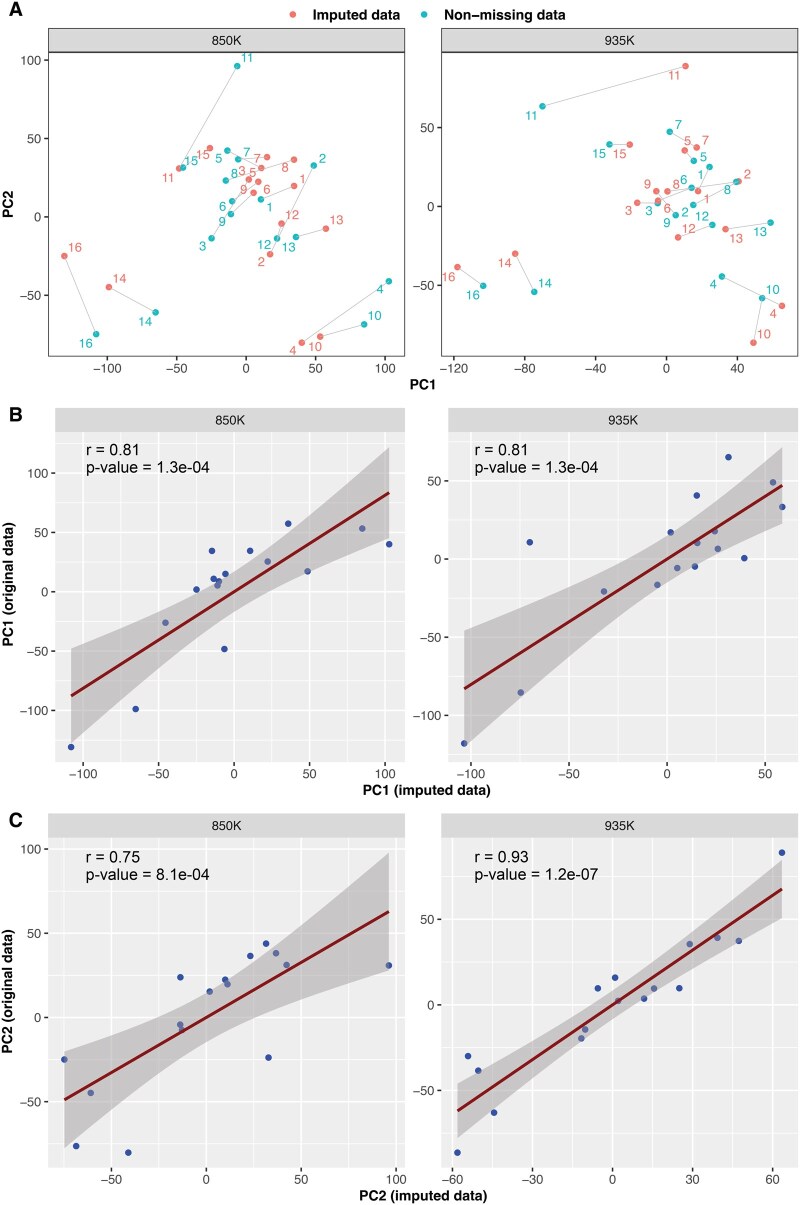
Evaluation of MOREL-DNN performance in imputing array-specific probes between Infinium MethylationEPIC v1.0 (850K) and Infinium MethylationEPIC v2.0 (935K). (A) Two-dimensional PCA projections based on original CpGs (array-specific CpGs, blue dots) and imputed CpGs (array-absent CpGs, red dots) for 850K dataset (left) and 935K dataset (right). The 16 brain tumor samples are labeled numerically from 1 to 16. (B) Correlation between the first principal component (PC1) derived from imputed CpGs (x-axis) and original CpGs (y-axis) for 850K dataset (left) and 935K dataset (right). (C) Correlation between the second principal component (PC2) derived from imputed CpGs (x-axis) and original CpGs (y-axis) for 850K dataset (left) and 935K dataset (right).

The results showed that, compared to array-specific data (i.e. the original beta values), the imputed data (i.e. beta values inferred from another array) closely preserved the original dimensionality across both datasets (Fig. 3A). Pearson correlation coefficients between PC1__original_ versus PC1__imputed_ were 0.81 for both 850K and 935K arrays (Fig. 3B). Similarly, the correlations between PC2__original_ and PC2__imputed_ were 0.75 and 0.93 for the 850K and 935K arrays, respectively (Fig. 3C). We also analyzed the 722 359 shared CpGs using the same approach applied to blood and GBM samples to assess MOREL’s imputation performance between the 850K and 935K arrays. The results demonstrated high accuracy (MAE = 0.0460–0.0505, Table 3, available as supplementary data at *Bioinformatics Advances* online). Collectively, these findings confirmed that MOREL effectively reconstructs missing methylation landscapes while preserving the intrinsic data structure and biological complexity.

### 3.4 Cross-platform imputation: RRBS versus mouse methylation arrays (MMA)

RRBS has been the predominant approach for profiling DNA methylation in mouse samples. Recently, the Illumina Mouse Methylation BeadChip (MMA) has emerged as a cost-effective and scalable alternative for large cohort studies. This shift introduces a similar challenge of integrating data across different platforms. To evaluate MOREL’s performance in this context, we analyzed 22 144 CpGs shared between the MMA and RRBS datasets from 37 mouse samples (Fennell *et al.* 2022). We randomly introduced 50% missing values into MMA data and RRBS data, respectively, to evaluate the imputation accuracy under two scenarios: imputing MMA data from RRBS and imputing RRBS data from MMA. The results showed that all four MOREL methods achieved high accuracy in both directions (Table 4, available as supplementary data at *Bioinformatics Advances* online); however, imputing MMA values from RRBS was slightly more accurate (MAE = 0.0377–0.0408) than imputing RRBS values from MMA (MAE = 0.0410–0.0470). These results demonstrate that MOREL is not limited to data generated from the same platform (e.g. 850K versus 935K arrays) but can effectively address cross-platform imputation challenges.

### 3.5 Rescuing missing CpGs for DNA methylation aging clocks

**Figure 4:**
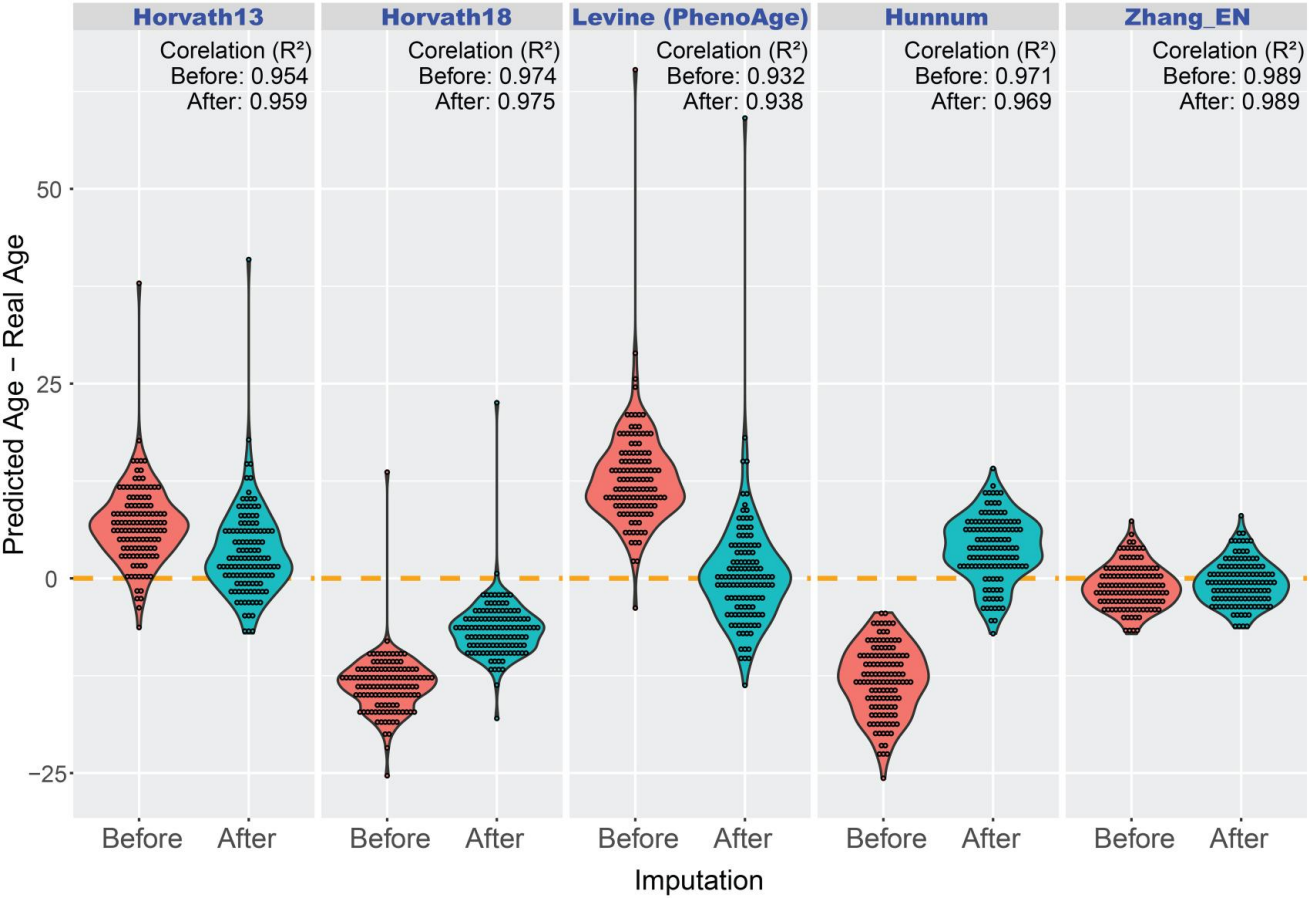
Imputation of missing CpG sites improves the accuracy of epigenetic age estimation across multiple epigenetic clocks. Bean plots show age prediction errors for 124 healthy human control samples using five published epigenetic clocks (Horvath13, Horvath18/Skin & Blood clock, PhenoAge, Hannum, and Zhang_EN) originally trained on 450K or 850K methylation arrays. Prediction error (y-axis) was calculated as the difference between predicted epigenetic age and chronological age for each individual. Because each clock includes CpG sites absent from the newer 935K array, initial predictions (“Before”) exhibited reduced accuracy. After imputing missing CpGs using MOREL, re-estimated epigenetic ages (“After”) showed markedly improved accuracy and increased correlation coefficients (R²) across all clocks.

DNA methylation-based epigenetic clocks rely on specific feature CpG sites whose methylation levels correlate with chronological age or other phenotypic traits. However, most published clocks were trained on earlier array versions, limiting their applicability to the latest 935K array. To evaluate MOREL’s ability to enhance clock performance, we analyzed 935K data from 124 healthy human control samples from a bipolar disorder study (unpublished) using five publicly available clocks—Horvath13 (Horvath 2013), Horvath18 (Horvath *et al.* 2018), PhenoAge (Levine *et al.* 2018), Hannum (Hannum *et al.* 2013), and Zhang_EN (Zhang *et al.* 2019)—originally developed on 450K or 850K arrays. As expected, each clock had a subset of CpGs absent from the 935K array. To assess the impact of imputation, we first estimated the epigenetic age using the original 935K data, then re-estimated the age after restoring missing CpGs with imputed values. All five clocks showed markedly improved accuracy after imputation (Fig. 4), demonstrating that recovering missing CpGs enhances the predictive performance of epigenetic clocks on newer methylation arrays.

### 3.6 Processing time and memory usage

The MOREL framework builds training models using all available data that do not exhibit structural missingness, leading to a linear increase in processing time and memory usage as more data are included during training. Processing time varies across models, with KNN being the fastest, followed by SVR, while DNN and RF require significantly more time.

For imputing 5% missing values in a dataset of 32 samples, KNN takes 15, 101, and 1104 s, whereas RF requires 834, 4413, and 30 033 s across 10 000, 50 000, and 250 000 CpGs, respectively. Memory usage also scales predictably, increasing from 3.57 GB at 10 000 CpGs to 9.29 GB at 50 000 CpGs and 37.68 GB at 250 000 CpGs (Table 5, available as supplementary data at *Bioinformatics Advances* online). These results demonstrate that MOREL scales predictably with dataset size while maintaining reasonable computational demands for large-scale DNA methylation studies.

## 4 Discussion

The Illumina EPIC arrays are widely used for genome-wide DNA methylation profiling across various research fields, generating vast amounts of data that have been deposited in repositories such as Gene Expression Omnibus (GEO). However, intrinsic incompatibilities between array versions hinder the full utilization of these valuable legacy datasets.

The primary goal of this study is to “restore” missing methylation data using state-of-the-art machine learning approaches. While our methods were developed and tested specifically on EPIC array-based DNA methylation data, they could also be applied to methylation data generated from RRBS, WGBS, cell-free DNA, and single-cell assays where missing values are also prevalent. Given that the human genome contains approximately 28 million CpGs, imputing DNA methylation values presents a greater computational challenge than imputing gene expression. To address this issue, splitting CpGs by chromosome significantly reduces computation time without compromising imputation accuracy.

Proximal CpG sites often exhibit correlated DNA methylation levels (co-methylation). Currently, our imputation approach does not consider the physical distance between CpGs. As a next step, we plan to incorporate genomic coordinates of CpGs along with those of candidate cis-regulatory elements (cCREs), including promoters, enhancers, CpG islands, and insulators, as defined by ENCODE. With over 300 000 cCREs identified by the ENCODE consortium, integrating these features into the training model may further enhance imputation accuracy but will require substantial computational resources.

Another limitation of the MOREL framework is that it currently supports imputation between only two datasets at a time (e.g. imputing 850K probes from 935K or vice versa) and cannot directly accommodate scenarios involving three or more data modalities, such as 850K, 935K, and RRBS. A practical workaround is to perform iterative imputation—for instance, first imputing between the EPIC arrays (850K versus 935K), followed by imputing the combined 850K + 935K dataset against RRBS.

## Supplementary Material

vbag052_Supplementary_Data

## Data Availability

HumanMethylation450 BeadChip data from 30 human whole blood samples are available from the Gene Expression Omnibus (GEO) under accession GSE105018. HumanMethylation450 data from 30 glioblastoma multiforme samples were obtained from The Cancer Genome Atlas via the Genomic Data Commons Data Portal. Illumina EPIC v1.0 (850K) and EPIC v2.0 (935K) methylation array data from 16 brain tumor samples are available from GEO (GSE229715). Mouse RRBS data and mouse methylation BeadChip data are available from ArrayExpress under accessions E-MTAB-12214 and E-MTAB-11985, respectively. Illumina EPIC v2.0 (935K) methylation data from 124 healthy control samples in the bipolar disorder study are unpublished and will be made available upon request.
